# Quantifying resistance and resilience to local extinction for conservation prioritization

**DOI:** 10.1002/eap.1989

**Published:** 2019-08-28

**Authors:** Lynda Donaldson, Jonathan J. Bennie, Robert J. Wilson, Ilya M. D. Maclean

**Affiliations:** ^1^ Environment & Sustainability Institute University of Exeter Penryn Campus Cornwall TR10 9FE United Kingdom; ^2^ Wildfowl & Wetlands Trust Slimbridge GL2 7BT United Kingdom; ^3^ Department of Geography University of Exeter Penryn Campus Cornwall TR10 9FE United Kingdom; ^4^ College of Life and Environmental Sciences University of Exeter Exeter EX4 4PS United Kingdom; ^5^ National Museum of Natural Sciences (MNCN‐CSIC) Madrid 28006 Spain

**Keywords:** colonization, conservation planning, *Cyperus papyrus*, extinction, habitat fragmentation, landscape, metapopulation, resilience, resistance, wetland

## Abstract

Species‐focused conservation planning is often based on reducing local extinction risk at key sites. However, with increasing levels of habitat fragmentation and pressures from climate change and overexploitation, surrounding landscapes also influence the persistence of species populations, and their effects are increasingly incorporated in conservation planning and management for both species and communities. Here, we present a framework based on metapopulation dynamics in fragmented landscapes, for quantifying the survival (resistance) and reestablishment of species populations following localized extinction events (resilience). We explore the application of this framework to guide the conservation of a group of threatened bird species endemic to papyrus (*Cyperus papyrus*) swamps in East and Central Africa. Using occupancy data for five species collected over two years from a network of wetlands in Uganda, we determine the local and landscape factors that influence local extinction and colonization, and map expected rates of population turnover across the network to draw inferences about the locations that contribute most to regional resistance and resilience for all species combined. Slight variation in the factors driving extinction and colonization between individual papyrus birds led to species‐specific differences in the spatial patterns of site‐level resistance and resilience. However, despite this, locations with the highest resistance and/or resilience overlapped for most species and reveal where resources could be invested for multispecies persistence. This novel simplified framework can aid decision making associated with conservation planning and prioritization for multiple species residing in overlapping, fragmented habitats; helping to identify key sites that warrant urgent conservation protection, with consideration of the need to adapt and respond to future change.

## Introduction

Global biodiversity is declining at an unprecedented rate (Newbold et al. 2015), yet the resources available to counteract this loss are finite and insufficient to ensure that ambitious global biodiversity targets are met (McCarthy et al. 2012). Establishing protected areas, defined by the Convention on Biological Diversity (CBD) as geographic locations that are “designated or regulated and managed to achieve specific conservation objectives,” is one of the main approaches for the safeguarding of biodiversity. The importance of these sites is recognized globally, with signatories to the CBD aiming to safeguard 17% of terrestrial land and inland water by 2020 as part of the Aichi Biodiversity Targets (CBD 2011). Priority sites such as Important Bird and Biodiversity Areas (IBAs; Fishpool and Evans 2001) and Alliance for Zero Extinction Sites (Ricketts et al. 2005), have been developed to ensure efforts are directed toward the most important locations for biodiversity. The designation and management of such areas is focused around boosting populations at individual sites to secure survival (Geldmann et al. 2013). However, landscapes are becoming increasingly fragmented as a result of changing land use practice (Newbold et al. 2015), while pressures are growing from climate change (Urban 2015) and overexploitation (Millennium Ecosystem Asessment 2005, WWF 2014). As a result, species may not remain within individual designated sites indefinitely, and the surrounding landscape context will likely play a key role for the regional persistence of species.

Reserve design and management have been influenced much by the theories of island biogeography and metapopulation dynamics (Diamond 1975, Hanski 1994a, Akcakaya et al. 2007). The principles associated with these theories state that long‐term persistence is dependent on balancing the processes of local extinction and colonization within sites across the landscape (Hanski 1998). In general, populations residing in small and/or low quality sites are at greater risk of extinction, while poorly connected sites are unlikely to be recolonized should extinction occur (MacArthur and Wilson 1967, Hanski 1999, Thomas et al. 2001). These concepts have been pivotal for site‐based design and more recently the establishment of landscape‐scale conservation initiatives (see Donaldson et al. 2017), though deciding which sites to invest in is complex (Hannah 2008) and remains the focus of much research (Whytock et al. 2018). Although it was originally suggested that various species respond similarly to local and landscape‐scale drivers of extinction and colonization such as habitat fragmentation (Hanski 1994a), this remains untested (Whytock et al. 2018). Considering the rapid rates of habitat loss and degradation worldwide, combined with limited budgets to combat such threats, finding efficient ways to identify and protect the key sites that sustain multiple species is paramount.

In essence, the factors influencing the importance of an individual site for the regional or landscape‐scale conservation of a species can be partitioned into (a) the resistance of the local population to extinction (Lawler 2009, Lake 2013), and hence the chances that the population persists through unfavorable periods or is able to act as a source for the (re)colonization of other sites; and (b) the resilience of the population to disturbance (Holling 1973, Lake 2013). Although the definition is still disputed (e.g., see Oliver et al. 2015), in the context of metapopulation dynamics, resilience can refer to the chances that a site will be recolonized following local extinction i.e., the process of “recovery” following a disturbance (Hodgson et al. 2015). Quantifying resistance and resilience from this perspective will prove a useful tool for conservation planning, ensuring that sites designated for conservation are not only robust to change, but have the capacity to bounce back from change should local extinction occur (Lawler 2009, Nimmo et al. 2015).

We apply this framework for determining landscape‐scale resistance and resilience to a group of bird species endemic to papyrus (*Cyperus papyrus*) swamps in East and Central Africa. Papyrus swamp is a highly fragmented habitat that has been experiencing rapid loss and degradation over recent decades, primarily from drainage and encroachment for commercial and subsistence agriculture (Maclean et al. 2011b, van Dam et al. 2014). This has led to the decline in populations of specialist bird species (Maclean et al. 2014) and the inclusion of some of these species on the IUCN Red List (IUCN 2017). Papyrus swamps are recognized as a regional conservation priority but as yet receive little protection (Fanshawe and Bennun 1991, Kipkemboi and van Dam 2016), and evidence for where protected areas should be designated is scarce. Conventional approaches toward the safeguarding of biodiversity within these swamps are based on their current occupancy: sites hosting high numbers of birds, for example, are regarded as priority areas for conservation (Maclean et al. 2011b). However, this fails to recognize either the resistance of individual sites, their resilience to unfavorable environmental extremes or changes to management, and their sensitivity to the persistence of other sites within the larger network. Papyrus swamps are exposed to frequent disturbances (Maclean et al. 2003a, 2006), seasonal drainage (Zsuffa et al. 2014), and will likely be subject to altered hydrology as the climate changes (Terer et al. 2012a). As a result, safeguarding a network of sites, where occupied sites can act as source populations for those subject to deterministic or stochastic extinction (Akcakaya et al. 2007), will help ensure populations can bounce back from disturbances that lead to localized population declines or extinctions. With multiple species using the same landscape, an understanding of the main factors that influence the population establishment and survival of each species, and the implications of any notable differences between species, is desirable for the identification of important sites.

Here, we use occupancy data for five species of papyrus‐endemic passerines collected from a network of swamps in southwest Uganda, to determine the local and landscape effects that influence extinction and colonization for each species. We then map the predicted probabilities of survival and colonization for each patch across the network and use this to draw inferences about the locations and landscapes that contribute most to regional resistance (meaning “survival”) and resilience (here denoting “colonization”) for each species, and whether there is spatial congruence in these among species. We conclude by compiling this information for all the study species, to establish the potential of overlapping priority sites that would ensure resistance and resilience for specialist species in the network, and discuss the wider application of this framework for conservation planning and prioritization.

## Methods

### Study system

Papyrus swamps host a suite of endemic passerines with distributions largely focused around parts of East and Central Africa (Maclean et al. 2014). This study focused on five such species: White‐winged Swamp‐warbler (*Bradypterus carpalis*), Greater Swamp‐warbler (*Acrocephalus rufescens* race *foxi*), Papyrus Canary (*Crithagra koliensis*), Papyrus Yellow Warbler (*Calamonastides gracilirostris*), and Carruthers's Cisticola (*Cisticola carruthersi*). All species are primarily restricted to papyrus, although papyrus yellow warbler and Carruthers's Cisticola are also known to inhabit wetland dominated by other vegetation types, namely *Miscanthidium* and *Typha* spp., when closely associated with papyrus (Vande weghe 1981). Previous work has shown that White‐winged Swamp‐warbler, Carruthers's Cisticola, and Papyrus Yellow Warbler preferentially inhabit the wetland interior, while the remaining two species are more often associated with swamp edge (Britton 1978, Donaldson et al. 2016). The species are also likely to differ in dispersal propensity (see *Analyses*).

Research was conducted across a network of papyrus swamps surrounding Lake Bunyonyi, Uganda (01°17′ S 29°55′ E). High densities of papyrus are found in this area, growing along deep valley bottoms and along the lake edge. The presence of some of the papyrus‐specialist birds has led to the designation of an IBA at the far north of the lake (BirdLife International 2017), while others have also been proposed (Maclean et al. 2014). All patches of papyrus swamp were located using a combination of 1:50000 topographical maps (Department of Land and Surveys, Entebbe), satellite imagery (Google Earth), local knowledge, and examination from motorboat and on foot. Following preliminary observations, a habitat patch was defined as wetland approximately >20 m long and >5 m wide suitable for breeding birds, separated by >10 m from other patches. Swamps dominated by other wetland vegetation types (here termed “broad wetland vegetation”) were included in the study for the two species inhabiting this habitat type. Carruthers's Cisticola was also found in areas of wetland recently converted to agriculture in this area (Donaldson et al. 2016). Shoreline fringing patches were surveyed for the presence of greater swamp‐warbler and papyrus canary, as preliminary observations over the 2 yr confirmed that only these species were ever located within this patch type.

### Data collection

Data were collected over two consecutive years (2014–2015) from 232 papyrus swamps, 287 shoreline fringing papyrus patches and 177 broad wetland patches (including papyrus). All patches were visited at least once per year by the same observer during the main breeding season (May–August), and the presence or absence of each species recorded. Surveys were conducted between ~06.45 and ~13.45 when the birds are most vocal, using intermittent playback to aid detection. Time spent surveying varied with patch size, ranging from a minimum of 5 min for small, low quality shoreline fringing patches, to a maximum of 7 h 15 min for large broad wetland patches (Appendix S1: Table S1). All of the study species are highly vocal, and almost always readily detectable within short periods of visiting the site (Maclean et al. 2006). To provide more formal evidence of detectability, we examined relationships between likelihood of detection and survey effort (Appendix S1), which highlight that the probability of incorrectly recording a species as absent when present during an average survey, was relatively small (Appendix S1: Fig. S1).

On the day of survey, coordinates were recorded from the edge of swamps in the UTM projection system using a handheld GPS unit (GPSMAP 64; Garmin, Lenexa, Kansas, USA), and sketch maps of the swamp were drawn to scale using topographical maps. Four distinct vegetation categories were assigned based on vegetation height and composition (Table 1 and see Muthuri et al. 1989, Maclean et al. 2006, Terer et al. 2012b, Donaldson et al. 2016) and the proportion of each estimated at all sites. Maps were digitized in ArcGIS v 10.1 (ESRI, Redlands, California, USA; UTM 35S) and used to estimate patch size, circularity (defined using the formula 4π area/perimeter^2^) and nearest edge distances between patches.

**Table 1:** Vegetation categories defined for papyrus swamp and broad wetland^a^

Vegetation category	Description	Age	Density	Typical height	Culm thickness	Senescence?
Disturbed wetland	cleared (harvested, burned), immature and/or regrown papyrus^a^, agricultural wetland^b^	0–1 yr	none (cleared)‐high (regrown)	low (0–2 m) to high (> 2 m)	thin	none
Moderately disturbed wetland	mature papyrus previously disturbed and fully regrown to maturity	>1 yr	moderate	high (>2 m)	thick	some
Undisturbed wetland	mature papyrus, not likely to be disturbed, any disturbance over 1.5 yr ago	>1.5 yr	low	high (>2 m)	thick	yes
Mixed vegetation wetland	mixed wetland vegetation containing > 40% papyrus^a^, poor growing conditions for papyrus	>1 yr	low	low (0–2 m)	thin	some

*Notes:* See Donaldson et al. (2016) for further details.

a Includes wetland dominated by other wetland types for two species also found in these areas (Carruthers's Cisticola and Papyrus Yellow Warbler; Maclean et al. 2006).

b Applicable to Carruthers's Cisticola only.

### Analyses

Two sets of analyses were undertaken to investigate the potential drivers of (1) patch colonization (determining “resilience”) and (2) the survival of populations within patches (as a proxy for “resistance”). All analyses were performed in R version 3.3.1 (R Core Team 2016) using generalized linear models with a binomial error distribution and logit link function. The response variable was the presence or absence of each species in year 2 (2015). Models of colonization were conducted on patches in which the species was absent in year 1 (2014), and either present (1, colonized) or absent (0) from those patches in year 2. Models of survival were based on patches where the species was present in year 1, and either absent (0, local extinction) or present (1, survival) in year 2.

Explanatory variables in both sets of analyses involved local and landscape factors from data collected in 2015. Relative patch size was similar between years (Pearson *R* ^2 ^= 1.0; Appendix S2: Table S1) and as the relative proportion of disturbed habitat per patch differed over the study period (papyrus *R* ^2 ^= 0.3, broad wetland *R* ^2 ^= 0.2; Appendix S2: Table S1), using habitat data collected in year 2 enabled us to most accurately capture the change in occupancy that occurred over the one year examined. Local variables analyzed were patch size (ha), patch circularity, and the proportion of three distinct vegetation categories: disturbed wetland, undisturbed wetland, and mixed wetland vegetation (Table 1). To avoid over‐fitting models, which would result if the sum of all categories is always one, moderately disturbed wetland was excluded from the analysis (see also Donaldson et al. 2016). Landscape variables comprised a measure of the functional connectivity of patch *i* as described by (Hanski 1994b):

(1) $$Si\left(t\right)=\sum p_{j}\exp\left(-\alpha d_{\mathrm{ij}}\right)A_{j}^{b}$$

where *p*_*j*_ is the occupancy of patch *j* in year 1 (*t*), α is a parameter that defines the dispersal kernel, *d*_*ij*_ is the nearest edge distance of the focal patch *i* to other patches *j*,* A*_*j*_ is the carrying capacity of patch *j,* usually approximated by area and *b* is a scaling function for patch emigration (*i ≠ j*). The parameter α was estimated for each species using the Markov chain Monte Carlo technique available in SPOMSIM software version 1.0 (Moilanen 2004): Greater Swamp‐warbler = 0.204, Papyrus Canary = 0.190, Carruthers's Cisticola = 0.070, White‐winged Swamp‐warbler = 0.021, Papyrus Yellow Warbler = 0.001. In metapopulation models, *A*_*j*_ is typically defined as patch area, as a proxy for population size (Ozgul et al. 2006). However, as shown in Donaldson et al. (2016), the density of birds at each site varies depending on a variety of other factors in addition to patch size. Thus, the density of all species was predicted at each site using the model averaged coefficients obtained in Donaldson et al. (2016) from point count survey data, and weighted by multiplying by patch size as an estimate of the *relative* population size for each species within each patch (*A*_*j*_). The parameter *b* was set to 1, assuming that emigration is proportional to abundance.

Exploratory analysis was conducted to determine the importance of intermediate levels of each vegetation type, as papyrus endemics can benefit from moderate disturbance (e.g., see Maclean et al. 2006, Donaldson et al. 2016). Models containing each individual vegetation category (disturbed wetland, undisturbed papyrus, and mixed vegetation) as linear predictors were tested against models that also contained each predictor as a squared term. The squared terms were subsequently retained in the global model when the Akaike Information Criterion (AIC) value obtained from the model including this term was lower than without (Burnham and Anderson 2002). The MuMIN package in R (Barton 2014) was used to create all possible combinations of the global model, including any relevant squared terms for the survival and colonization data sets (Appendix S3: Table S1). Models were ranked by AIC_c_ (AIC corrected for small sample size) and a set of models within ΔAIC_c_ ≤ 2 of the top model created for each species (Burnham and Anderson 2002). Model averaging was performed across all models within the top ranked set to obtain parameter estimates, and the relative importance (RI) of each term within the top set was recorded (Burnham and Anderson 2002, Johnson and Omland 2004). Full model averaged coefficients were used to predict the probability of colonization and survival of each patch for each species across the network, based on patch data collected from the 2015 survey. Semivariograms of the residuals from the predicted vs observed values for each data set were created using the geoR package in R (Ribeiro and Diggle 2001), to ensure there was no evidence of spatial structure in our models.

**Figure 1:**
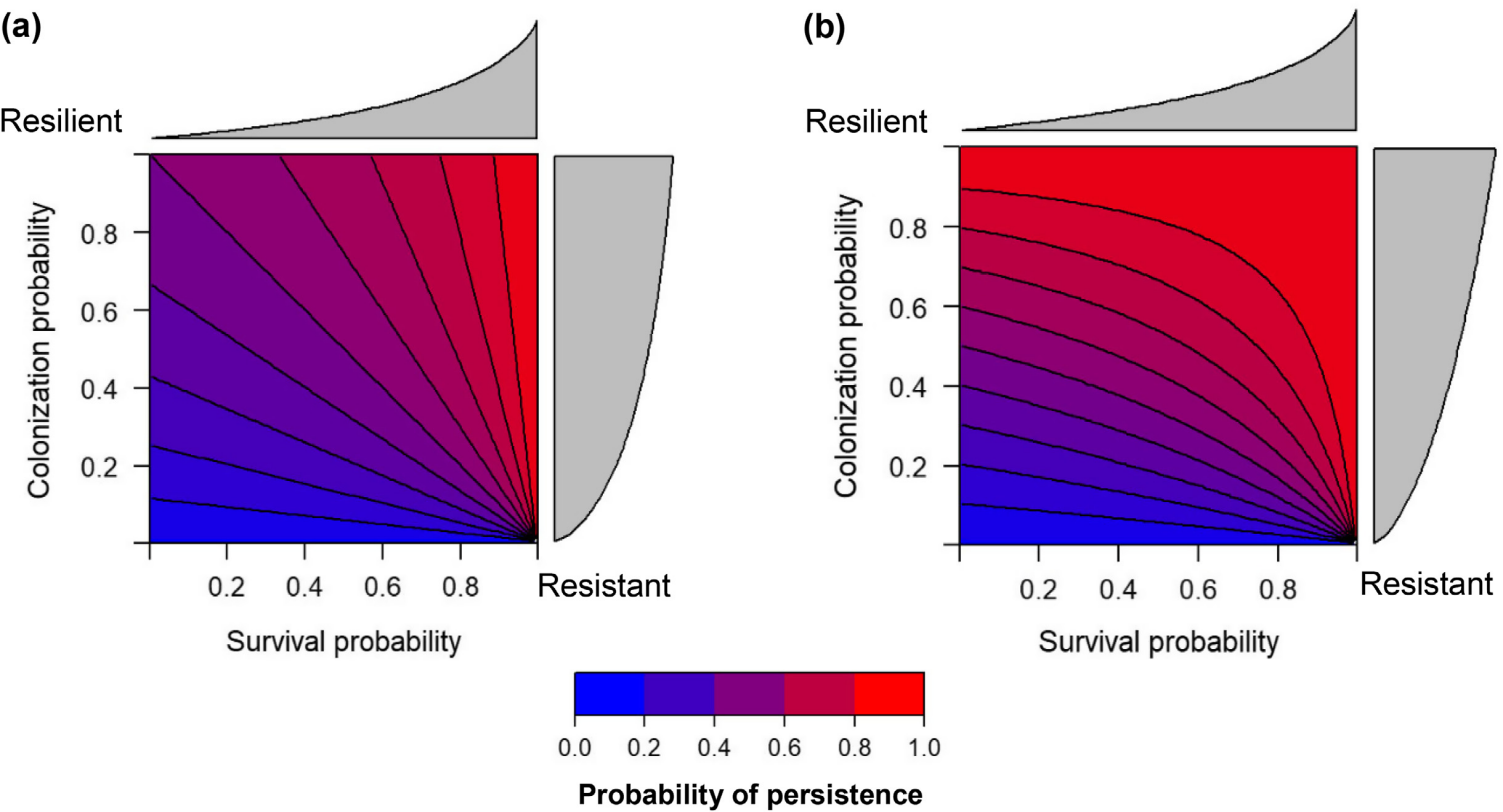
Relationship between inter‐annual probability of survival and colonization on long‐term steady‐state persistence. In (a), rescue effects, whereby new populations can be established by colonizers in the same year as an existing population goes extinct, are assumed not to occur. In (b), rescue effects are assumed to occur.

Data were collected over two consecutive years, yet most conservation decisions are made over longer time frames. In theory, any swamp in which a species has an annual survival probability < 1 will eventually lose that species without further recolonizations, and any swamp with a colonization probability > 0 will eventually be colonized. It is thus necessary to consider how the balance of colonizations and extinctions translate into steady‐state probability of a species persisting in any given swamp. Assuming no rescue effect (i.e., new populations are not established by colonists in the same year as an existing population goes extinct), steady‐state persistence (*P*) is given as follows:

(2) $$\frac{C}{1-S+C}$$

where *C* is annual probability of colonization and *S* is annual probability of survival. Here, *P* increases linearly with *C* and *S* and exceeds 0.5 provided *S + C *>* *1 (Fig. 1a). Alternatively, where new populations may be established by colonists in the same year as an existing population goes extinct, steady‐state persistence (*P*) is given as follows:

(3) $$\frac{C}{1-S+SC}.$$

Here, *P* is not linearly related to *C*, and *S* is disproportionately influenced by colonization probability and can exceed 0.5 even when *S + C *<* *1 (Fig. 1b). Work by Britton (1978) shows that the egg laying period of papyrus endemics typically coincides with the main rainy season, though breeding can occur outside this period, and the situation in which rescue effects occur is thus more likely. Irrespective of the assumptions made about rescue effects, patches with a higher probability of colonization can be classed as more “resilient” than those with a lower chance of colonization, while patches with a relatively high probability of survival represent sites with a higher level of “resistance” than those with a high chance of local extinction (Fig. 1a, b). At present, patches with a relatively high chance of both survival (if occupied) and recolonization (if unoccupied), can be both resistant and resilient to extinction, and therefore have a high probability of persistence over time. Conversely, patches with a relatively low chance of both survival (if occupied) and colonization (if unoccupied), have low resistance and resilience (and thus low long‐term steady‐state persistence), and can be considered “marginal” (Lawson et al. 2012) (Fig. 1a, b). All patches and their corresponding status were mapped across the network to recognize areas of importance for regional persistence.

Finally, the capacity to conserve multiple species was determined using overlapping maps of resistance and resilience for each species. Since multiple papyrus patches were often located within larger broad wetland sites, the predictions for the two broad wetland species (Papyrus Yellow Warbler and Carruthers's Cisticola) for a given wetland were allocated to those papyrus patches within that particular swamp, in order for the networks for all species to be directly comparable. Similarly, shoreline fringing patches were marked as “marginal” for the three species that did not use these patches, on the overlapping plots only.

## Results

### Patch survival and colonization for papyrus endemic birds

The number of patches colonized between 2014 and 2015 ranged from 3 for White‐winged Swamp‐warbler to 69 for Greater Swamp‐warbler (Table 2; Appendix S4: Fig. S1). All species were more likely to colonize large swamps (relative importance = 1; see Appendix S3: Table S2 for all output for colonization analyses described), though patch size was not classed as significant (whereby 95% confidence intervals do not cross 0) for White‐winged Swamp‐warbler (RI = 0.34). The probability of colonization was higher in more connected patches for Greater Swamp‐warbler (RI = 1) and Carruthers's Cisticola (RI = 1), but this term was not found in the top model set for White‐winged Swamp‐warbler or Papyrus Yellow Warbler and did not significantly influence colonization for Papyrus Canary (RI = 0.28). More circular patches were more likely to be colonized by Carruthers's Cisticola (RI = 1), and White‐winged Swamp‐warbler (RI = 1), but not by Papyrus Canary (RI = 0.71), Greater Swamp‐warbler (RI = 0.15), or Papyrus Yellow Warbler (RI = 0.12). For all species studied, patch colonization was not significantly influenced by the proportion of disturbed or undisturbed wetland. Both variables were found in a small number of models within the top set, but did not have high relative importance, with the exception of Papyrus Canary (undisturbed vegetation; RI = 1). The probability of colonization was positively affected by the proportion of mixed papyrus vegetation for White‐winged Swamp‐warbler only (RI = 1).

**Table 2:** Presence–absence survey data for suitable patches for each species from 2014–2015

Species	Patches surveyed	Colonized	Survived	Extinct	Vacant
GSW	519^a^	69	206	63	181
PC	519^a^	44	40	16	419
WWW	232	3	41	12	176
CC	160^b^,^c^	8	31	4	117
PYW	177^b^	10	17	3	147

*Note:* Species are Greater Swamp‐warbler (GSW), Papyrus Canary (PC), White‐winged Swamp‐warbler (WWW), Carruthers's Cisticola (CC), Papyrus Yellow Warbler (PYW).

a Includes shoreline fringing patches.

b Includes broad wetland vegetation.

c Includes agricultural wetland.

The number of local extinction events ranged from three for Papyrus Yellow Warbler and four for Carruthers's Cisticola, to 63 for Greater Swamp‐warbler (Table 2; Appendix S4: Fig. S1). All species were more likely to survive in large patches, although this was not significant for Carruthers's Cisticola (RI = 1) or Papyrus Yellow Warbler (RI = 0.87; see Appendix S3: Table S3 for all outputs of the survival analyses described). Population survival was also more likely in less circular patches for Greater Swamp‐warbler (RI = 1), and in more circular patches for White‐winged Swamp‐warbler (RI = 1). As with colonization, the level of disturbance within a patch was not a good predictor of survival for any of the species. Disturbed wetland was only in the top set for Greater Swamp‐warbler (RI = 0.42) and Papyrus Yellow Warbler (RI = 0.28), while undisturbed wetland was in the top model set for Greater Swamp‐warbler (RI = 0.93), White‐winged Swamp‐warbler (RI = 0.19), and Papyrus Yellow Warbler (RI = 0.3). The proportion of mixed papyrus within a patch negatively influenced the chance of survival for three of the species, shown to be marginally significant for Papyrus Canary (RI = 1), but not significant for Greater Swamp‐warbler (RI = 1) or White‐winged Swamp‐warbler (RI = 0.17). Finally, the probability of survival within a patch was not influenced by connectivity for any of the species (Greater Swamp‐warbler RI = 0.26; Papyrus Canary RI = 0.28; White‐winged Swamp‐warbler RI = 0.22; Carruthers's Cisticola RI = 0.55).

### Predicted turnover across networks

**Figure 2:**
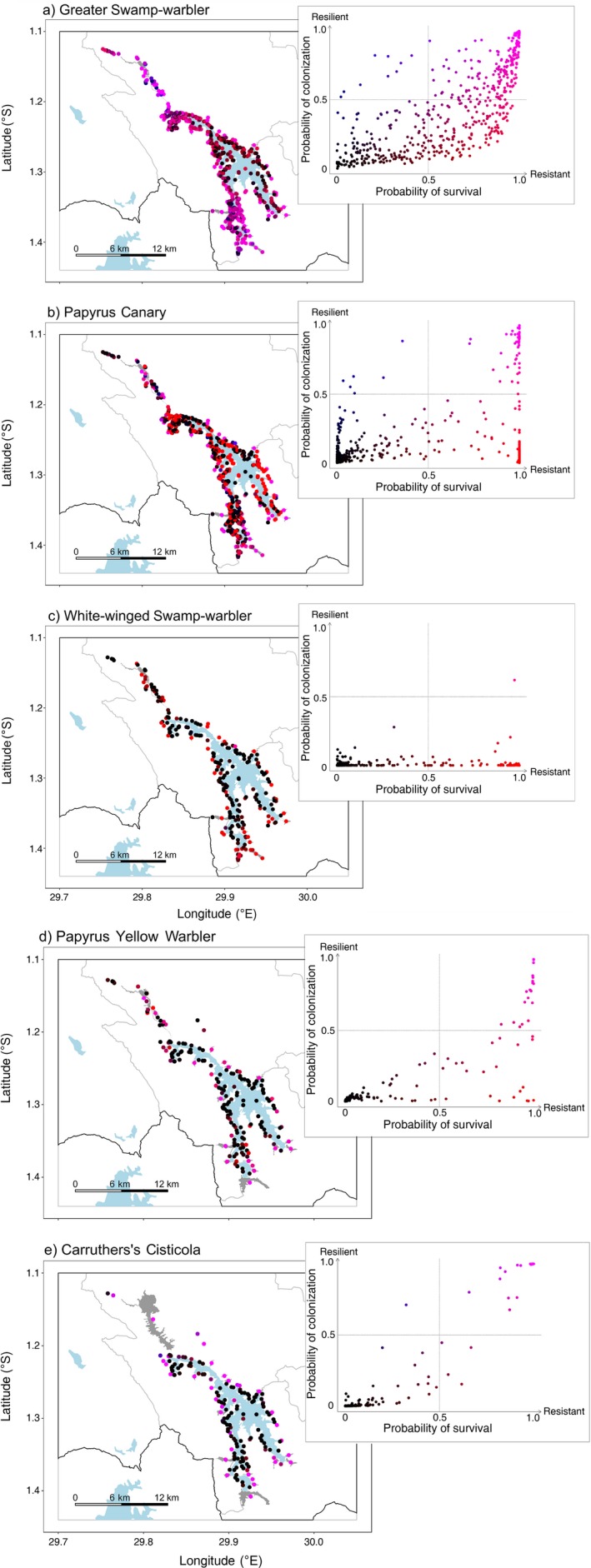
Maps of predicted probabilities of colonization to and survival in each patch for the five study species (a–e) at Lake Bunyonyi, Uganda. Points show the center coordinates of each patch, colored by the strength of relationship between survival and colonization. Inset: probabilities of survival and colonization for individual habitat patches, showing the color coding of patches used on the maps (blue, high probability of colonization, low survival; red, high probability of survival, low colonization; purple, high probability of colonization and survival; black, low probability of colonization and survival). Suitable wetland is shown in gray. Occupancy data over 2014–2015 is displayed in Appendix S4: Fig. S1.

The proportion of patches in the network with high probabilities of both survival and colonization varied between species (Fig. 2a–e). These were distributed throughout the network for all species, though strongholds were apparent in the far north and south of the lake for Greater Swamp‐warbler, while the only patch predicted to have a relatively high chance of survival and colonization for White‐winged Swamp‐warbler was located in the center. In contrast, all species were predicted to have a very high number of patches with a low probability of either colonization or survival (Fig. 2a–e). These were well spread throughout the network, most notably along the central edges of the lake.

Comparing patches with relatively low levels of each process, considerably more patches had a low probability of colonization (low resilience) than survival (low resistance). No patches for White‐winged Swamp‐warbler (Fig. 2c) or Papyrus Yellow Warbler (Fig. 2d) had a low probability of survival with a high chance of colonization, while very few patches were predicted to lie on this side of the continuum for Carruthers's Cisticola (Fig. 2e), Papyrus Canary (Fig. 2b), and Greater Swamp‐warbler (Fig. 2a). Patches with a lower probability of survival were often located close to highly resistant and resilient patches for all species. Patches with relatively low probabilities of colonization alone, were generally located toward the center of the network for Greater Swamp‐warbler (Fig. 2a) and Papyrus Canary (Fig. 2b), clustered toward the north and south of the lake for Papyrus Yellow Warbler (Fig. 2d), and spread throughout the network for White‐winged Swamp‐warbler (Fig. 2c).

### Overlapping priorities

**Figure 3:**
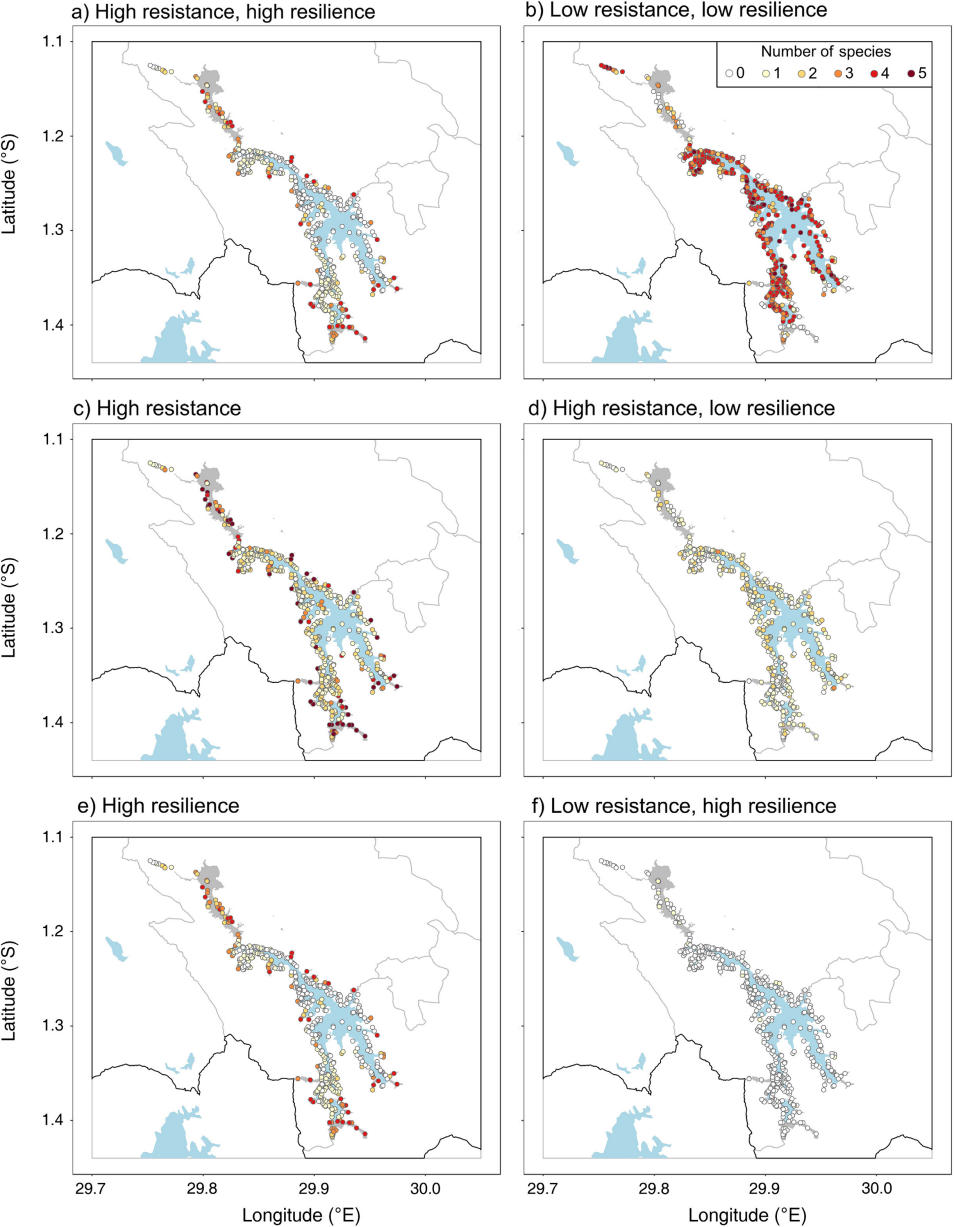
Maps displaying the predicted categories and level of overlap for all species across the network at Lake Bunyonyi, Uganda, based (for illustrative purposes) on 0.5 probability of survival (high resistance) and colonization (high resilience): (a) high resistance, high resilience, (b) low resistance, low resilience, (c) high resistance (with high and low resilience), (d) high resistance, low resilience, (e) high resilience (with high and low resistance), (f) low resistance, high resilience. Key: 0, no species within corresponding category; 1+ is the number of species within corresponding predicted category for specified patch. See Fig. 1 for explanation of categories.

Mapping the predicted categories for all species together (Fig. 3a–f) illustrated that the most overlap between all five species existed between patches with high levels of resistance (including high and low probabilities of colonization; Fig. 3c), and low levels of both resistance and resilience (Fig. 3b), while patches with low resistance and high resilience intersected the least (Fig. 3f).

There were many more patches classified as resistant for all five species (Fig. 3c), than patches classified as resilient (Fig. 3e). These were situated along the length of the lake, with some clusters around the larger swamps at the north and south of the study area; corresponding closely to those sites that were both resistant and resilient for four of the study species (Fig. 3a). Patches with lower resilience but high resistance only overlapped for up to three species (Fig. 3d), while patches with low resistance and high resilience did not coincide for any of the study species (Fig. 3f). Those patches with relatively low resistance and resilience for all five species were located around the edge of the lake, consisting primarily of the fringing shoreline patches (Fig. 3b).

## Discussion

Despite being closely related species with similar habitat requirements, there is a degree of variation in the local and landscape characteristics driving the processes of population survival and reestablishment in papyrus‐endemic birds. However, using a framework based on the probabilities of survival and colonization from one year to the next, we have identified that several parts of the landscape are still important for the resistance and resilience of all species combined. This is a promising approach for conservation decision making and prioritization in fragmented landscapes where urgent protection of key sites is required, and demonstrates the role of quantifying extinction and colonization for informing multispecies conservation plans.

### Regional persistence of multiple species

Enhancing persistence is one of the key objectives associated with the establishment of protected sites (Margules and Pressey 2000). To achieve this goal, conservation planning often focuses on ensuring population survival, yet understanding the processes that affect colonization is also important for the recovery of populations faced with extinction (Davies et al. 2005). Within fragmented landscapes, the persistence of a metapopulation is dependent on the balance of rates of extinction and colonization (Hanski and Gilpin 1991). Understanding the drivers of these processes is a significant step in conservation planning (Franzén and Nilsson 2010, Robles and Ciudad 2012), enabling the identification of the parts of the landscape that host particular species (Hodgson et al. 2011). However, the importance of different local and landscape characteristics remains unknown for numerous species (Whytock et al. 2018), leading to uncertainty regarding the variation that exists for multiple species occupying the landscape (Hodgson et al. 2009). Should different approaches give rise to drastically different outcomes (Brooks et al. 2006), for example, developing conservation strategies that are suitable for all in need would be considerably more challenging.

The response of population survival and establishment to habitat and landscape characteristics does vary among specialist species of passerines residing in a network of papyrus swamps. Although they are within the same guild, variation exists between the ecological characteristics of these species, such as habitat preferences (Vande weghe 1981) and capacity for dispersal. Local extinction is most closely linked with patch, rather than landscape‐scale variables (Lawson et al. 2012), particularly patch size and quality, because of their influence on population carrying capacities (Hanski 1999, Thomas et al. 2001). In line with this, all species were less likely to become extinct in larger swamps, while aspects associated with habitat quality for these species (Donaldson et al. 2016) were often found to be predictors for survival. The more elusive White‐winged Swamp‐warbler, for example, had a lower chance of extinction in more circular swamps with a low edge : area ratio, while Greater Swamp‐warbler, an edge species (Britton 1978, Donaldson et al. 2016), had a higher probability of survival in swamps with a higher edge : area ratio. Further, the three species most closely associated with papyrus were less likely to occur in mixed papyrus (Donaldson et al. 2016), whereas the likelihood of occurrence of the broader wetland species was not impacted by this habitat type.

In contrast to survival, colonization is often linked with landscape variables, namely connectivity (Hanski et al. 1996), driven by the distance between patches, matrix habitat, dispersal ability, and the number of potential dispersers (Dorp and Opdam 1987). Previous work by Maclean et al. (2006) found no correlation between patch occupancy and proximity to neighboring swamps, though the range of distances examined were far greater than in the present study, and it is likely that the majority of isolated swamps lay beyond the dispersal distance of the species studied. Connectivity influenced colonization for those estimated to show relatively lower levels of dispersal (Carruthers's Cisticola and Greater Swamp‐warbler), while in species with higher dispersal capabilities, or that are known to feed outside of swamps (e.g., Papyrus Canary; Britton 1971), colonization probability was largely unaffected by levels of connectivity at the scale of this study. Enhancing connectivity is often assumed to be a fundamental element of conservation planning, without any prior investigation (Hodgson et al. 2009). However, our results caution against simply focusing on connectivity for the benefit of all species. Over recent years, the role of area and quality in driving the process of colonization has been recognized (Franzén and Nilsson 2010, Glorvigen et al. 2013, Bohenek et al. 2017). Large patches are considered more likely to be detected by the disperser (Vos et al. 2000), and can be purposely selected by active compared to passive dispersers (Glorvigen et al. 2013), which could explain why the majority of species here were more likely to colonize larger swamps. Patch quality is also a significant influencer of habitat selection (Robles and Ciudad 2012, Glorvigen et al. 2013), hence why many of the habitat factors known to influence quality (Donaldson et al. 2016) were also in the top set for colonization in this study.

### Resistance vs. resilience across a network

Despite developments from metapopulation theory, there is still a tendency in conservation planning to focus efforts on individual sites. Recognition of multiple sites is rarely explicitly considered (Gaston et al. 2008), yet allowing the landscape to function as a network is crucial in order to support biodiversity over the long‐term (Lawton et al. 2010). In modern landscapes, where habitat fragmentation is the norm (Tilman et al. 2017), ensuring that populations are both resistant and resilient to extinction is axiomatic (Lawler 2009, Hodgson et al. 2015). By recognizing the mechanisms that drive these aspects, planners can identify the most important parts of the landscape (Nimmo et al. 2015), and note what is likely to be restricting the ability of a species to persist now, or how species could respond to changing land use in the future (Opdam et al. 1995).

Applying this resistance–resilience framework to papyrus‐endemic birds, we identified that multiple sites within the network did have relatively low levels of resilience compared to resistance. Regardless of rescue effects, these patches are unlikely to support the persistence of populations over time (Fig. 1a, b). Specialist species are often assumed to possess poor abilities to colonize sites, compared to more generalist species (Davies et al. 2005). Indeed, the species most closely associated with papyrus inhabited more patches with a low chance of colonization but not survival within their network than the broad wetland species, which generally had more habitat available to colonize. In turn, any future changes to the habitat of these species that cause extinction within parts of the network, such as seasonal drainage or wide‐scale habitat disturbance (Maclean et al. 2003a, Zsuffa et al. 2014), could be catastrophic for regional population persistence. With no flow of individuals from outside these sites, these patches effectively act as sink populations (Pulliam 1988), which may fail to exist over the long‐term (Hansen and Rotella 2002). Since most species rely on large patches for colonization, as landscape fragmentation and loss continues to increase (Tilman et al. 2017), levels of resilience will continue to decline, even as the need for (re‐)colonizations increases (Hanski and Gilpin 1991, Whytock et al. 2018). This scenario of low resilience throughout the network is especially a concern for White‐winged Swamp‐warbler, which had virtually no patches that would likely be colonized following an extinction event (Fig. 2c).

### Prioritizing conservation effort

Strategic conservation planning is vital to ensure that the limited time and money available for conservation is channeled most effectively (Brooks et al. 2006). Numerous methods have been developed to assist with this process (Margules and Pressey 2000), but the uncertainty surrounding where to invest remains (Whytock et al. 2018). Alongside guaranteeing the persistence of individual species, protected sites also strive to be representative of biodiversity as a whole (Margules and Pressey 2000). Thus, not only are we faced with the challenge of ensuring sites are resistant and resilient to change, but we must apply this approach to multiple species residing in the same landscape (Darwall and Vié 2005). Given the increasing pressures from growing human populations and acute shortage of land (Tilman et al. 2017), particularly in developing regions, conservation planning must also consider what is practically achievable in modern landscapes when setting conservation priorities in the real world, aside from population viability alone (Donaldson et al. 2017).

Mapping the probability of survival and colonization for multiple papyrus passerines at Lake Bunyonyi highlights that, as it stands, a number of swamps are relatively resistant and/or resilient for all species combined (Fig. 3a, c, e) and thus have a high probability of steady‐state persistence (Fig. 1a, b). Similarly, there are numerous sites for which the likelihood of survival and establishment are comparatively low for all the study species (Fig. 3b), and therefore are unlikely either to be resistant or resilient, with relatively little chance of any of these species’ persisting in these patches over the longer term (Fig. 1a, b). Thus, if the focus for conservation is on the preservation or protection of key existing sites that offer resistance and resilience, achieving this for multiple species is possible. Moreover, as it is impractical to conserve all swamps for biodiversity in this region (Maclean et al. 2014), and given the limited resources and challenges of enforcing existing wetland policy (Kipkemboi and van Dam 2016), overlapping “marginal” sites could potentially be regarded as lower priority for conservation (Lawson et al. 2012) and enable more intensive use of some wetland sites by local people. However, this would necessitate observation of the consequences for the species in the network as a whole; examining the role of these neglected patches as stepping stones to promote gene flow between populations (Gibbs 2001), for example, as well as ensuring that the remaining sites are adequately managed and monitored to maintain their levels of resilience.

In contrast, papyrus patches with lower probabilities of either colonization or survival showed very little interspecific overlap (Fig. 3d, f). As a result, restoring habitats with a view to improve either of these aspects on its own (Bulman et al. 2007) is unlikely to yield results for all species collectively, and resources would have to be spread thinly to reverse any limitations for all. Restoring wetlands has been suggested as a mechanism to reverse the devastation caused to papyrus swamps over the past few decades (Morrison et al. 2012, Kiwango et al. 2013); enabling the continued provision of ecosystem services to local communities (van Dam et al. 2011), as well as benefiting the wildlife reliant on it. However, much of the drained land has been converted to cropland to maintain production and mediate the effects of population growth (Carswell 2002, Terer et al. 2012a), and reversing this will likely impose high social and economic costs for those depending on these areas for their livelihoods. In the Kigezi region of Uganda, most wetland areas suitable for cultivation have already been drained, thus the ability to maintain food security in this area has likely reached its limit (Carswell 2002). Previous work by Maclean et al. (2003b, 2011a) highlighted that draining swamps for agriculture is less profitable than preserving swamps in order to enable the continuation of the multifunctional services they provide (Donaldson et al. 2016), particularly for the rural poor. As a result, placing priority on limiting habitat loss at existing swamps (van Dam et al. 2014) and ensuring that important sites for the persistence of biodiversity are offered at least some protection, will concurrently benefit people who receive the most value from the continued existence of these wetlands for their livelihoods (Maclean et al. 2011a). Disturbance by local people for subsistence use did not impact the ability of patches to survive or be colonized in this study, suggesting that the activities of local people could continue in moderation in those key sites highlighted as important for the persistence of papyrus endemics.

Wetlands in East Africa generally suffer from lack of cohesion in policy and the failure of parties to adhere to any guidance in place (Kipkemboi and van Dam 2016). However, Uganda operates a decentralized governance whereby the management of wetland functions is carried out at the local level of villages and parishes, which has already proved effective for implementing policies more locally (Maclean et al. 2011a, 2014). Since the main drivers of wetland loss across the region are poverty and income inequity and commercial reclamation (Maclean et al. 2011a), devolving power to the local level where the benefit obtained from the presence of swamps is far greater, could be an effective structure for others across East Africa to employ and ultimately assist with the conservation of remaining key wetland sites in urgent need of protection.

## Conclusion

This study shows how an understanding of the mechanisms that lead to the survival and establishment of populations can be used to offer insight into the levels of resistance and resilience for multiple species residing across fragmented landscapes. Although slight differences in the response to various habitat characteristics existed between species, mapping the predicted dynamics of these species does show that there are multiple sites likely to be relatively resistant and resilient to extinction for all species combined. Incorporating this landscape‐scale resistance‐resilience framework into conservation planning can help inform the allocation of valuable resources, with consideration of the growing need for biodiversity to respond and recover to future change.

## Data Availability

Data are available on Figshare: https://doi.org/10.6084/m9.figshare.8969810.v1
