# Supplementary material for: Quantifying resistance and resilience to local extinction for conservation prioritization
Source: Ecol Appl. 2019 Aug 28;29(8):e01989. doi: 10.1002/eap.1989 (PMC6916261; doi:10.1002/eap.1989)
Supplement: Supplementary file 1 [file EAP-29-na-s001.pdf]

Supporting Information. Donaldson, L., Bennie, J.J., Wilson, R.J. and Maclean, I.M.D. 2019. Quantifying resistance and resilience to local extinction for conservation prioritization *Ecological Applications.*

Appendix S1: Likelihood of detection of papyrus-specialist birds

In order to highlight the probability of detecting each of the five study species during presence-absence surveys, additional data was collected on the frequency of detection within a subset of swamps surrounding Lake Bunyonyi, Uganda. Given the need to quantify habitat associations and distributions across all sites within the breeding season, it was not feasible to conduct multiple visits across all sites during this research (e.g. Mackenzie et al. 2002; Royle et al. 2005). As an alternative, the amount of time spent at each patch during each survey over the two years was recorded, and the probability of detection was modelled over time in a subset of sites. In turn, the probability of detection could be quantified as follows:

Detection survey

Patches known to be occupied by at least one of the study species were visited between 07:00 and 13:30; within the survey period used for data collection. The number of observations per minute for each species (visual and/or by sound) were recorded over a period ranging from 30 to 146 minutes, from 1 - 6 randomly selected locations at a subset of swamps. Each observation was treated as a separate encounter, regardless of the number of individuals recorded (see below). Playback was used intermittently throughout, representing a typical survey. In total, over 29 hours of this detectability data was collected from 23 different points across 11 papyrus (0.08 ha~54.5 ha) and 6 broad wetland patches (2.03 ha~311.5 ha).

Analysis

Total length of survey during the presence-absence data collection varied depending on the size of the patch. Thus, data collected for the detectability survey was separated into small (<2.1 ha) and large (>2.1 ha) patches, based on the typical time spent surveying patches of these sizes. The datasets collected per species during each individual survey were analyzed separately. To account for the temporal autocorrelation arising as a result of the tendency of birds to sing regularly for a few minutes before becoming silent again, data were analyzed using generalized estimating equations (GEEs) with a binomial error structure in R package geepack (Halekoh et al. 2006). Using this approach, clusters of temporally-autocorrelated data points can be incorporated into the framework to estimate the mean probability of a bird being detected per minute. In turn, using standard probability propagation formulae, the likelihood of detection over any time interval can be estimated as:

$$P_t = 1 - (1 - P_1)^t \quad [S1]$$

where P_t is the probability of detecting the bird during time t and P_1, the mean probability of detecting the bird per minute. In effect this offers a conservative approach to calculate detectability, since some of the delayed detections are likely to be "new" detections (i.e. a bird did newly arrive in that area of swamp) and detections are clustered into one-minute periods, regardless of the number of detections within that minute. Clusters were identified using the R package mclust (Fraley et al. 2012), which automatically specifies the optimal number of clusters and assigns each data point to a cluster using a Bayesian model comparison. No prior was assumed. The cumulative probability was subsequently estimated over 120 minutes for small and large patches. The probabilities of detection were then plotted separately for the individual surveys for each species against time, and compared with the average presence-absence survey time for which a species was recorded as absent.

Presence-absence survey data

Over the 2 years of data collection, presence-absence surveys were conducted for an average of 20 minutes for small papyrus patches, and up to an average of 164 minutes for large broad wetland swamps (Table S1). In small swamps, a species was recorded as absent following a mean of 14 minutes at patches for greater swamp-warbler, and a mean of up to 34 minutes for papyrus yellow warbler and Carruthers's cisticola. Meanwhile, large swamps were surveyed for a period of ~67 minutes for Carruthers's cisticola before marked as absent, and up to a mean of 98 minutes for papyrus yellow warbler. Greater swamp-warbler was not recorded as absent at any of the larger swamps over the 2 years of survey (Table S1).

Table S1. Mean survey time (minutes) spent at suitable small (<2.1 ha) and large (>2.1 ha) patches for all species during 2014-2015 presence-absence data collection. 'All patches' represents the mean time spent surveying all suitable patches for that species; 'Absent patches' shows the mean time spent at patches before that species was recorded as absent. Standard deviations are shown in brackets. n = total number of suitable patches surveyed within small and large categories.

**Species**	**Small patches (mins)**			**Large patches (mins)**		
	**n**	**All patches**	**Absent patches**	**n**	**All patches**	**Absent patches**
**Greater swamp-warbler**	487	20 (15)	14 (9)	32	130 (81)	N/A
**Papyrus canary**	487	20 (15)	18 (13)	32	130 (81)	86 (39)
**White-winged swamp-warbler**	200	29 (19)	27 (17)	32	130 (81)	77 (26)
**Papyrus yellow warbler**	141	34 (24)	34 (24)	36	150 (91)	98 (43)
**Carruthers's cisticola**	130	35 (25)	34 (25)	30	164 (95)	67 (9)

Probability of detection

Results suggest that most species had >95% probability of detection within the average time spent surveying a patch before it was marked as absent (Fig. S1). Within smaller swamps, Carruthers's cisticola and white-winged swamp-warbler had the highest chance of detection within the mean survey time, while papyrus canary had the lowest probability of being detected during the time spent surveying (Fig. S1a). This is likely due to the low densities of these species in smaller patches, being relatively less vocal, and the fact they have been known to occasionally forage outside of swamps (Vande weghe 1981). Detectability within large swamps was notably higher, particularly within the longer time periods spent at these sites (Fig. S1b; Table S1). The results show that the detectability of Carruthers's cisticola was slightly lower in two (13%) of the surveys for larger swamps (Fig. S1b), due to the fact that densities in these patches were relatively lower than some of the other sites surveyed here.

Overall, these results confirm the high likelihood of detection of the study species within the average presence-absence survey time. We recognize that there is a chance of false absences for some swamps within the network, particularly in smaller patches where species may move through more frequently. However, the analysis performed here is a conservative approach, and combined with previous research which demonstrated that surveying for more extensive periods made little difference to the records of these species (Maclean et al. 2006),

we can be confident that the chances of missing a bird in a given patch during our surveys was relatively low.

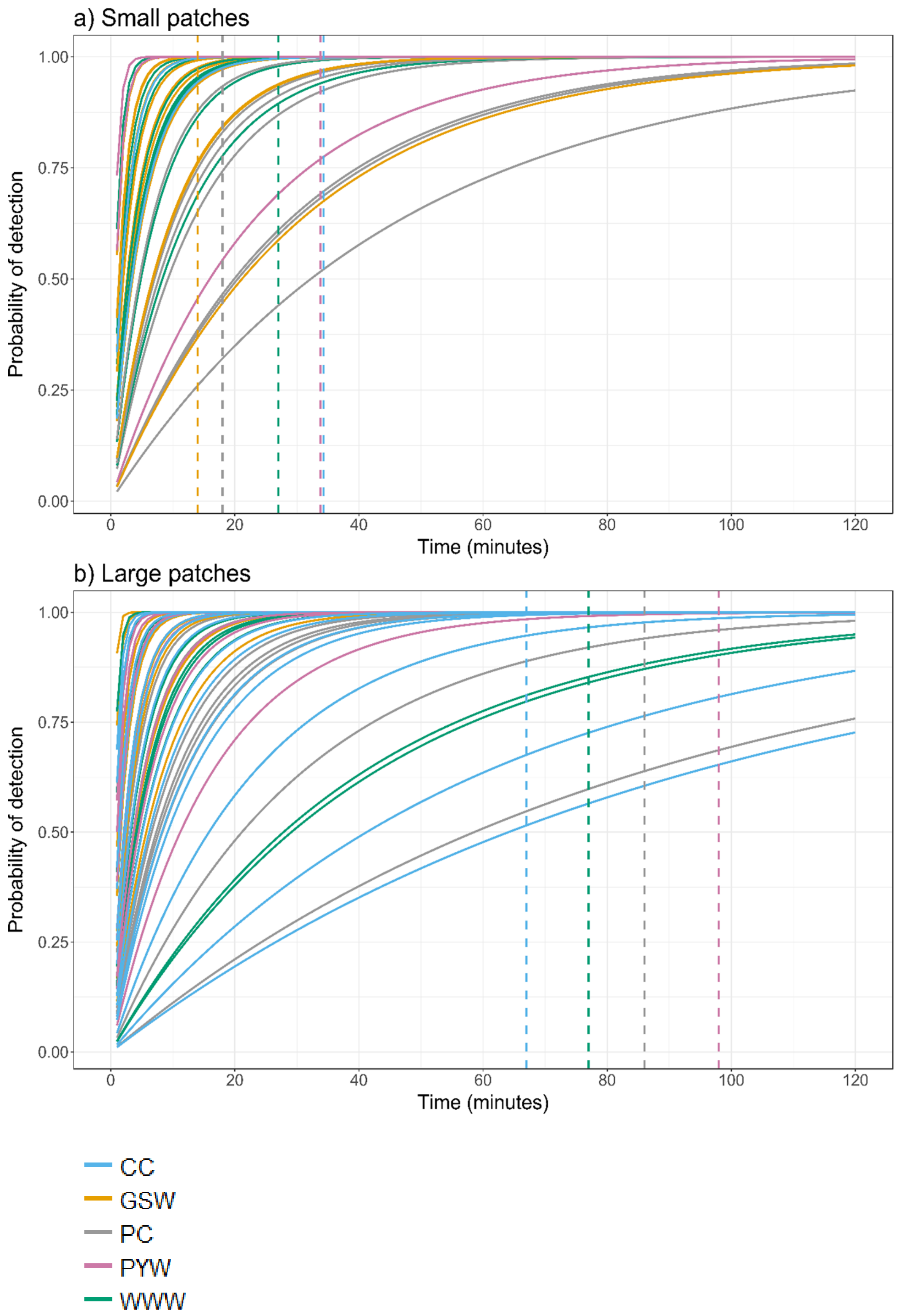

Fig. S1. Probability of detection for each species within a) small patches (<2.1 ha) and b) large patches (>2.1 ha) over 120 minutes. Solid colored lines represent the probability of detection for each species during each of the detection surveys (n = number of *detection*

surveys in wetland areas containing that species): CC = Carruthers's cisticola (a: n=3, b: n=15), GSW= greater swamp-warbler (a: n=11, b: n=10), PC = papyrus canary (a: n=9, b: n=10), PYW = papyrus yellow warbler (a: n=3, b: n=8), WWW = white-winged swamp-warbler (a: n=8, b: n=11). Dashed colored lines indicate the mean presence-absence survey time for patches where that species was recorded as absent over the 2 years of survey (see also Table S1).

Literature cited

Fraley, C., T. Adrian E. Raftery, B. Murphy, and L. Scrucca. 2012. mclust Version 4 for R: Normal Mixture Modeling for Model-Based Clustering, Classification, and Density Estimation Technical Report No. 597. Department of Statistics, University of Washington.

Halekoh, U., S. Højsgaard, and J. Yan. 2006. The R Package geepack for generalized estimating equations. Journal of Statistical Software 15:1–11.

Mackenzie, D. I., J. D. Nichols, G. B. Lachman, S. Droege, J. A. Royle, and C. A. Langtimm. 2002. Estimating site occupancy rates when detection probabilities are less than one. Ecology 83:2248–2255.

Maclean, I. M. D., M. Hassall, R. R. Boar, and I. R. Lake. 2006. Effects of disturbance and habitat loss on papyrus-dwelling passerines. Biological Conservation 131:349–358.

Royle, J. A., J. D. Nichols, and M. Kéry. 2005. Modelling occurrence and abundance of species when detection is imperfect. Oikos 110:353–359.

Vande weghe, J.-P. 1981. L'avifaune des papyraies au Rwanda et au Burundi. Le Gerfaut 71:489–536.
