# Supplementary material for: Quantifying resistance and resilience to local extinction for conservation prioritization
Source: Ecol Appl. 2019 Aug 28;29(8):e01989. doi: 10.1002/eap.1989 (PMC6916261; doi:10.1002/eap.1989)
Supplement: Supplementary file 2 [file EAP-29-na-s002.pdf]

Supporting Information. Donaldson, L., Bennie, J.J., Wilson, R.J. and Maclean, I.M.D. 2019. Quantifying resistance and resilience to local extinction for conservation prioritization *Ecological Applications.*

Appendix S2: Change in patch size and disturbance over survey period

Table S1. Mean patch size (ha) and proportion of disturbed habitat in 2014 and 2015 for all wetland patches surveyed across the study site.

**Wetland type**	**Patch area (ha)**		**Proportion of disturbed wetland**	
	**2014**	**2015**	**2014**	**2015**
**Papyrus**	1.22	1.06	0.23	0.17
**Broad wetland (Papyrus yellow warbler)**	3.75	3.19	0.14	0.11
**Broad wetland (Carruthers's cisticola)**[†]	6.58	5.73	0.14	0.10

[†]Includes agricultural wetland
