# Supplementary material for: Quantifying resistance and resilience to local extinction for conservation prioritization
Source: Ecol Appl. 2019 Aug 28;29(8):e01989. doi: 10.1002/eap.1989 (PMC6916261; doi:10.1002/eap.1989)
Supplement: Supplementary file 3 [file EAP-29-na-s003.pdf]

Supporting Information. Donaldson, L., Bennie, J.J., Wilson, R.J. and Maclean, I.M.D. 2019. Quantifying resistance and resilience to local extinction for conservation prioritization *Ecological Applications.*

Appendix S3: Colonization and survival analysis

Table S1. Global models for colonization and survival analysis for all species: greater swamp-warbler (GSW), papyrus canary (PC), white-winged swamp-warbler (WWW), Carruthers's cisticola (CC), papyrus yellow warbler (PYW)

Species	Colonization	Survival
**GSW**	log(area) + connectivity + circularity + disturbed + undisturbed + mixed veg + disturbed^2 + mixed veg^2 + undisturbed^2	log(area) + connectivity + circularity + disturbed + mixed veg + undisturbed + disturbed^2 + mixed veg^2 + undisturbed^2
**PC**	log(area) + connectivity + circularity + disturbed + undisturbed + mixed veg + undisturbed^2	log(area) + connectivity + circularity + disturbed + mixed veg + undisturbed + disturbed^2 + mixed veg^2
**WWW**	log(area) + connectivity + circularity + disturbed + undisturbed + mixed veg + disturbed^2 + mixed veg^2	log(area) + connectivity + circularity + disturbed + mixed veg + undisturbed + disturbed^2
**CC**	log(area) + connectivity + circularity + disturbed + undisturbed + mixed veg + mixed veg^2	log(area) + connectivity + circularity + mixed veg + undisturbed + disturbed
**PYW**	log(area) + connectivity + circularity + disturbed + undisturbed + mixed veg	log(area) + connectivity + circularity + disturbed + mixed veg + undisturbed

Table S2. Full model averaged output (ΔAICc ≤ 2) for colonization analysis (2014-15).

Species	Variable	Estimate	Std. Error	Lower CI	Upper CI	Significance	RI
GSW (n=13)	(Intercept)	-1.047	0.974	-2.966	0.872		
	**area (log)**	**0.732**	**0.179**	**0.380**	**1.083**	***	**1**
	**connectivity**	**0.044**	**0.010**	**0.025**	**0.062**	***	**1**
	mixed papyrus	-0.985	0.871	-2.696	0.727		0.78
	disturbed	-1.152	1.097	-3.307	1.004		0.73
	undisturbed	0.288	1.355	-2.375	2.950		0.59
	mixed papyrus ^{2}	-0.409	0.837	-2.052	1.234		0.29
	disturbed^2	-0.472	1.100	-2.632	1.688		0.26
	undisturbed^2	-0.682	1.597	-3.817	2.454		0.23
	circularity	-0.002	0.005	-0.012	0.009		0.15
WWW (n=5)	(Intercept)	-10.681	4.863	-20.300	-1.093	*	
	**circularity**	**0.099**	**0.054**	**-0.008**	**0.206**	•	**1**
	**mixed papyrus**	**105.624**	**61.976**	**-16.700**	**227.901**	•	**1**
	mixed papyrus^2	-455.681	335.511	-1120.000	206.371		1
	undisturbed	1.439	2.266	-3.020	5.896		0.46
	area (log)	0.248	0.541	-0.817	1.313		0.34
	disturbed	-0.395	2.102	-4.540	3.747		0.12
PC (n=9)	(Intercept)	0.300	1.003	-1.668	2.269		
	**area (log)**	**1.186**	**0.179**	**0.833**	**1.538**	***	**1**
	undisturbed	2.949	2.562	-2.082	7.980		1
	undisturbed^2	-4.798	2.985	-10.660	1.063		0.91
	mixed papyrus	-1.420	1.047	-3.476	0.635		0.83
	circularity	0.013	0.012	-0.010	0.037		0.71
	disturbed	-0.387	0.835	-2.025	1.252		0.31
	connectivity	0.006	0.013	-0.021	0.032		0.28
CC (n=3)	(Intercept)	-10.422	4.556	-19.436	-1.408	*	
	**area (log)**	**4.248**	**1.417**	**1.443**	**7.052**	**	**1**
	**connectivity**	**0.013**	**0.006**	**0.001**	**0.024**	*	**1**
	**circularity**	**0.098**	**0.047**	**0.004**	**0.191**	*	**1**
	disturbed	1.835	4.131	-6.307	9.976		0.3
	undisturbed	-0.272	1.399	-3.038	2.493		0.19
PYW (n=5)	(Intercept)	-2.268	0.912	-4.065	-0.472	*	
	**area (log)**	**1.230**	**0.285**	**0.666**	**1.793**	***	**1**

mixed papyrus	-0.767	1.369	-3.461	1.927	0.39
undisturbed	0.249	0.938	-1.598	2.097	0.16
disturbed	-0.484	2.257	-4.931	3.963	0.13
circularity	0.001	0.010	-0.018	0.021	0.12

Species: GSW = greater swamp-warbler; WWW = white-winged swamp-warbler; PC = papyrus canary; CC = Carruthers's cisticola; PYW = papyrus yellow warbler. n = number of models within the top model set (ΔAICc ≤ 2). CI = Confidence Intervals. Variables; RI = Relative Importance (proportion of models including this term); Levels of significance: *** p = 0.001, ** p = 0.01, * p =0.05, • p = marginal. ^{2}indicates squared term.

Table S3. Full model averaged output (ΔAICc ≤ 2) for survival analysis (2014-15).

Species	Variable	Estimate	Std. Error	Lower CI	Upper CI	Significance	RI
GSW	(Intercept)	4.935	1.075	2.821	7.049	***	
(n=10)	**area (log)**	**0.843**	**0.207**	**0.436**	**1.250**	***	**1**
	**circularity**	**-0.045**	**0.011**	**-0.066**	**-0.024**	***	**1**
	mixed papyrus	-3.143	2.161	-7.387	1.102		1
	undisturbed	3.848	2.551	-1.166	8.861		0.93
	undisturbed^2	-4.439	2.806	-9.953	1.075		0.87
	disturbed^2	-3.098	2.632	-8.267	2.071		0.71
	disturbed	-1.048	1.901	-4.780	2.683		0.42
	mixed papyrus^2	1.210	2.186	-3.083	5.504		0.36
	connectivity	0.003	0.007	-0.011	0.016		0.26
WWW	(Intercept)	-2.288	5.012	-12.255	7.678		
(n=4)	**area (log)**	**2.056**	**0.665**	**0.721**	**3.391**	**	**1**
	**circularity**	**0.062**	**0.034**	**-0.006**	**0.131**	•	**1**
	connectivity	0.003	0.010	-0.016	0.023		0.22
	undisturbed	-0.231	0.787	-1.799	1.337		0.19
	mixed papyrus	-0.340	1.398	-3.129	2.449		0.17
PC	(Intercept)	2.957	2.616	-2.268	8.181		
(n=2)	**area (log)**	**2.174**	**0.704**	**0.761**	**3.587**	**	**1**
	**mixed papyrus**	**-89.115**	**50.256**	**-189.931**	**11.701**	•	**1**
	mixed papyrus^2	339.036	239.775	-141.995	820.066		1
	connectivity	0.016	0.049	-0.081	0.113		0.28
CC	(Intercept)	-3.527	5.522	-14.602	7.549		
(n=2)	area (log)	2.650	1.583	-0.559	5.858		1
	connectivity	0.006	0.008	-0.010	0.021		0.55
PYW	(Intercept)	-3.619	3.212	-10.275	3.037		
(n=4)	area (log)	2.050	1.633	-1.324	5.425		0.87
	undisturbed	2.860	6.942	-11.391	17.111		0.3
	disturbed	14.506	35.648	-58.098	87.110		0.28
	circularity	0.010	0.043	-0.079	0.099		0.12

Species: GSW = greater swamp-warbler; WWW = white-winged swamp-warbler; PC = papyrus canary; CC = Carruthers's cisticola; PYW = papyrus yellow warbler. n = number of models within the top model set (ΔAICc ≤ 2). CI = Confidence Intervals. Variables; RI = Relative Importance (proportion of models including this term): Levels of significance: *** p = 0.001, ** p = 0.01, * p =0.05, • p = marginal. ^{2}indicates squared term.
