# Supplementary material for: Quantifying resistance and resilience to local extinction for conservation prioritization
Source: Ecol Appl. 2019 Aug 28;29(8):e01989. doi: 10.1002/eap.1989 (PMC6916261; doi:10.1002/eap.1989)
Supplement: Supplementary file 4 [file EAP-29-na-s004.pdf]

Supporting Information. Donaldson, L., Bennie, J.J., Wilson, R.J. and Maclean, I.M.D. 2019. Quantifying resistance and resilience to local extinction for conservation prioritization *Ecological Applications.*

Appendix S4: Patch occupancy maps

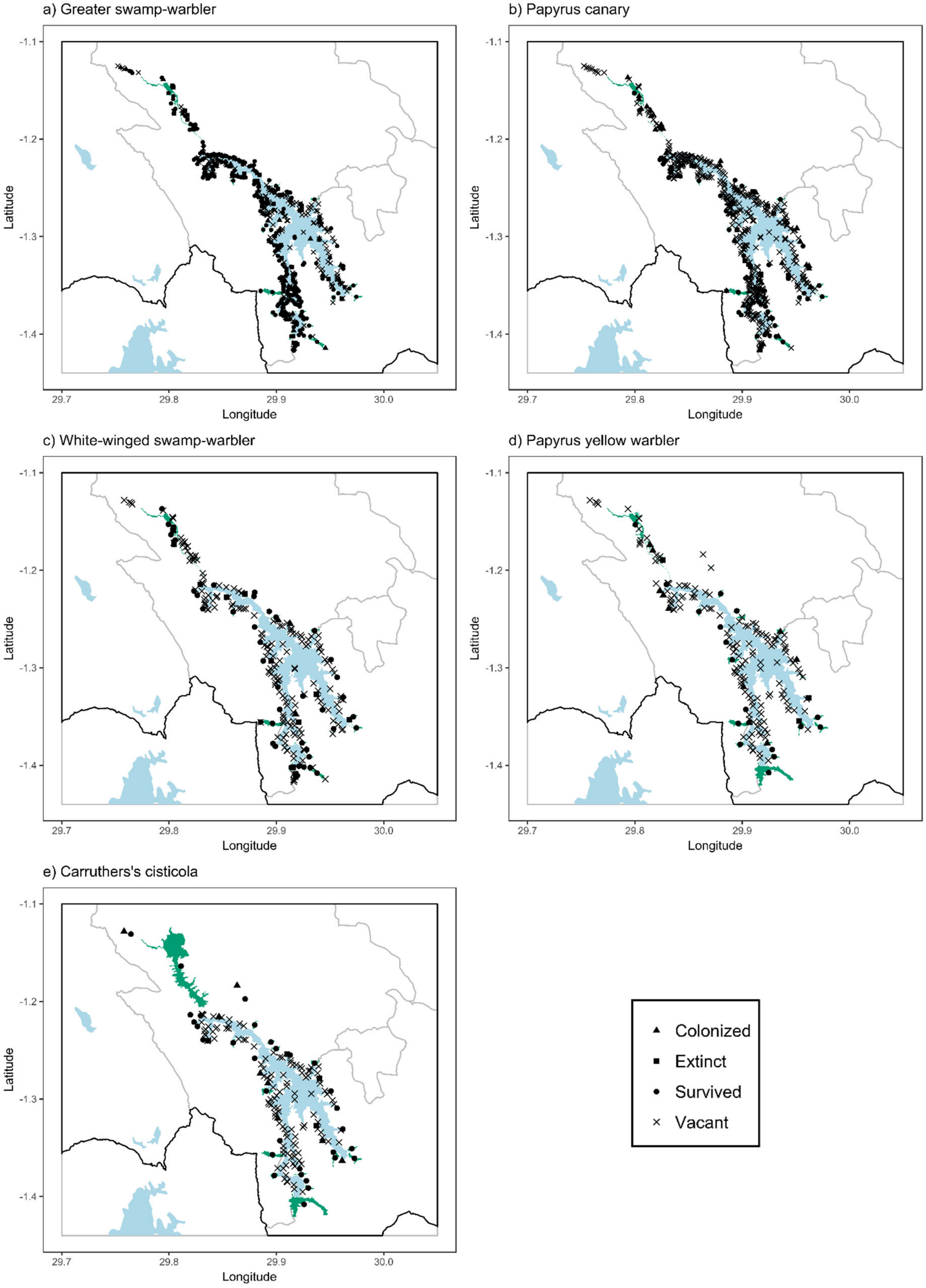

Fig. S1. Map of 2014-2015 occupancy data for five study species (a-e) at wetland patches (green) surrounding Lake Bunyonyi, Uganda.
